# Rhinoviral stimuli, epithelial factors and ATP signalling contribute to bronchial smooth muscle production of IL-33

**DOI:** 10.1186/s12967-015-0645-3

**Published:** 2015-08-29

**Authors:** Jenny Calvén, Hamid Akbarshahi, Mandy Menzel, Cemil Korcan Ayata, Marco Idzko, Leif Bjermer, Lena Uller

**Affiliations:** Division of Respiratory Immunopharmacology, Department of Experimental Medical Science, BMC D12, Lund University, 221 84 Lund, Sweden; Department of Pneumology, University Hospital Freiburg, Killianstrasse 5, 79106 Freiburg, Germany

**Keywords:** IL-33, dsRNA, Rhinovirus, Asthma, Bronchial smooth muscle cells, Bronchial epithelial cells, ATP

## Abstract

**Background:**

Bronchial smooth muscle cells (BSMCs) from severe asthmatics have been shown to overexpress the Th2-driving and asthma-associated cytokine IL-33. However, little is known regarding factors involved in BSMC production of IL-33. Rhinovirus (RV) infections cause asthma exacerbations, which exhibit features of Th2-type inflammation. Here, we investigated the effects of epithelial-derived media and viral stimuli on IL-33 expression in human BSMCs.

**Methods:**

Primary human BSMCs from healthy (n = 3) and asthmatic (n = 3) subjects were stimulated with conditioned media from primary human bronchial epithelial cells (BECs), double-stranded (ds)RNA, dsRNA/LyoVec, or infected with RV. BSMCs were also pretreated with the purinergic receptor antagonist suramin. IL-33 expression was analysed by RT-qPCR and western blot and ATP levels were determined in cell supernatants.

**Results:**

RV infection and activation of TLR3 by dsRNA increased IL-33 mRNA and protein in healthy and asthmatic BSMCs. These effects were inhibited by dexamethasone. BSMC expression of IL-33 was also increased by stimulation of RIG-I-like receptors using dsRNA/LyoVec. Conditioned media from BECs induced BSMC expression of IL-33, which was further enhanced by dsRNA. BEC-derived medium and viral-stimulated BSMC supernatants exhibited elevated ATP levels. Blocking of purinergic signalling with suramin inhibited BSMC expression of IL-33 induced by dsRNA and BEC-derived medium.

**Conclusions:**

RV infection of BSMCs and activation of TLR3 and RIG-I-like receptors cause expression and production of IL-33. Epithelial-released factor(s) increase BSMC expression of IL-33 and exhibit positive interaction with dsRNA. Increased BSMC IL-33 associates with ATP release and is antagonised by suramin. We suggest that epithelial-derived factors contribute to baseline BSMC IL-33 production, which is further augmented by RV infection of BSMCs and stimulation of their pathogen-recognising receptors.

## Background

Interleukin-33 (IL-33), a recently described member of the IL-1 cytokine family [1], has been forwarded as a potent driver of T-helper-2 (Th2)-type immunity and allergic inflammation [1–3]. Constitutive expression of IL-33 protein is predominantly found in nuclei of structural cell types, notably epithelial and endothelial cells [1, 4, 5]. Moreover, elevated levels of IL-33 have been demonstrated in situ in bronchial epithelium as well as bronchial smooth muscle cells from subjects with severe asthma, implicating IL-33 in asthma pathogenesis [6, 7]. Further support of this role comes from murine models [3, 8, 9] and large-scale genome-wide association studies in which asthma-susceptibility loci have been identified in the regions for *IL33* and the cognate receptor for IL-33, *IL1RL1* (i.e. ST2) [10, 11].

Release of IL-33 has principally been associated with cellular damage, proposing that IL-33 functions as an endogenous danger signal or alarmin [12]. However, a recent study demonstrated that exposure to the fungus *Alternaria Alternata* resulted in IL-33 secretion from bronchial epithelial cells in the absence of cellular necrosis and that this secretion was mediated via autocrine release of ATP and purinergic P2-receptor (P2R) activation [13]. At high concentrations, extracellular ATP is also considered an alarmin and is proposed to be involved in allergen-driven lung inflammation [14, 15]. Additionally, a pathophysiological role of ATP/P2R-axis in chronic lung disease is supported by findings of accumulated levels of ATP both in the airways of patients with asthma and chronic obstructive pulmonary disease (COPD) as well as in animal models of airway inflammation [14–18].

Rhinovirus (RV) infections are main triggers of asthma exacerbations [19, 20]. Double-stranded RNA (dsRNA) intermediates formed during RV replication initiate host innate immune responses via activation of pattern recognition receptors (PRRs), including toll-like receptor 3 (TLR3) and the retinoic acid-inducible gene-I (RIG-I)-like receptors RIG-I and melanoma-differentiation associated-gene 5 (MDA5) [21]. Previous studies have reported TLR3-induced IL-33 expression [22–24], but data regarding RV-induced IL-33 production and specific involvement of dsRNA-sensing receptors are currently limited [25]. Further, to our knowledge it has not been described if P2R signalling may have a role in TLR3-mediated IL-33 induction.

In the last decades, accumulated evidence supports an active role for bronchial smooth muscle cells (BSMCs) in immunomodulation and airway inflammation. Findings include expression of PRRs and secretion of a wide repertoire of cytokines and other mediators in response to external stimuli [26, 27]. We recently demonstrated that BSMCs express functional RIG-I-like receptors in addition to the previously described TLR3, and that activation of these receptors by viral stimuli results in BSMC production of type I and III interferons [28]. In the present study we hypothesized that BSMC expression of IL-33 may be induced by epithelial-derived factors as well as by viral stimuli. Hence, we have examined effects of bronchial epithelial conditioned media and of viral stimuli, including RV infection and specific ligands for TLR3 and RIG-I-like receptors on primary human BSMCs from healthy and asthmatic individuals.

## Methods

### Primary cell cultures

Primary human BSMCs from three healthy subjects and three subjects with mild to moderate asthma were commercially obtained from Lonza (Walkersville, MD, USA) as cryopreserved cells. BSMCs were cultured under standard conditions (5 % CO_2_ and 37 °C) in smooth muscle growth medium supplemented with SingleQuots and 5 % FBS (SmGM-2; Clonetics, San Diego, CA, USA) and used in experiments at passage 4–8. Primary human bronchial epithelial cell (BEC) cultures from four individuals with asthma were established from epithelial brushings obtained by bronchoscopy as previously described [29]. BECs were cultured under standard conditions in bronchial epithelial growth medium supplemented with SingleQuots (BEGM; Clonetics, San Diego, CA, USA) and used at passage 2–3 for the generation of BEC-derived conditioned medium. Ethical approval was obtained from the regional ethical review board at Lund University Sweden, and all study participants provided written informed consent.

### Generation of BEC-derived conditioned media

BECs were seeded into collagen coated 12-well plates (Nunc, Life Technologies, Carlsbad, CA, USA) and grown to confluency. The growth medium was then replaced with 1 ml/well of bronchial epithelial basal medium (BEBM; Clonetics) supplemented with 1 % insulin-transferrin-selenium and 0.1 % BSA. Some BECs were additionally treated with 10 μg/ml of the synthetic dsRNA analogue polyinosine-polycytidylic acid (Poly (I:C); InvivoGen San Diego, CA, USA). After 24 h incubation, control and dsRNA-generated conditioned media were subsequently collected, centrifuged to remove cell debris and stored in −80 °C until further use.

### Stimulation and RV infection of BSMCs

BSMCs were seeded into 12-well plates and when 80–90 % confluent, the growth medium was replaced with SmGM-2 containing reduced serum (0.5 % FBS) for 24 h before the cells were stimulated with BEC-derived conditioned media (diluted 1:100 to 1:10 in serum-reduced SmGM-2) alone or in combination with 10 μg/ml dsRNA [Poly (I:C)]. BEBM diluted 1:10 in serum-reduced SmGM-2 was used as vehicle control. Similarly, BSMCs were stimulated with a range of concentrations of dsRNA, dsRNA/LyoVec (Poly (I:C) in complex with the transfection agent LyoVec; InvivoGen), non-hydrolysable ATP (ATP-γ-S; Sigma-Aldrich, Gillingham, UK) or medium control (serum-reduced SmGM-2). Additionally, BSMCs were infected with human RV1B at different levels of multiplicity of infection (MOI) as previously described [28], treated with medium control or ultraviolet (UV)-inactivated RV (UVi-RV; non-infectious virus control) corresponding to the highest MOI used for RV infection. When indicated, 10 μg/ml chloroquine, 1 μg/ml dexamethasone or 10–100 μM suramin (all from Sigma-Aldrich) was added 1 h prior to stimulation or RV infection and was present until the end of the experiment. At the indicated time points, cell supernatants were collected for ATP and protein quantification and cells lysed and harvested for mRNA or protein expression analyses.

### IL-33 mRNA analysis by real-time qPCR

Total RNA was extracted from primary BSMCs, reverse transcribed and analysed with real-time qPCR as previously described [28]. The primer sequences used to detect IL-33 were supplied by Primerdesign (Southampton, UK) and were as follows: AAAGAAAGATAAGGTGTTACTGAGTTA (forward) and GCAACCAGAAGTCTTTTGTAGG (reverse). IL-33 was normalised to the geometric mean of two reference genes, ubiquitin c (UBC) and glyceraldehyde 3-phosphate dehydrogenase (GAPDH), and relative gene expression compared to control was analysed using the ΔΔCt method.

### Western blot analysis of IL-33 protein

Total protein was extracted from cell lysates using a sample buffer for western blot containing 1 % TritonX-100, 10 mM Tris–HCl, 50 mM NaCl, 5 mM EDTA, 30 mM sodium pyrophosphate, 50 mM NaF, 0.1 mM Na_3_VO_4_, and complete protease inhibitor cocktail (Sigma-Aldrich). Protein concentration was measured using BCA protein assay reagent kit (PIERCE ThermoScientific). Lysates were dissolved in Laemmli buffer, boiled and electrophoretically separated in a 12 % SDS-PAGE gel before transferred to an Immobilon-P Membrane, PVDF (Merck Millipore). Blots were blocked in 5 % (w/v) milk in Tris-buffered saline Tween-20 and incubated overnight at 4 °C with the primary antibody, anti-IL-33 antibody Nessy-1 clone (N1) (Enzo Life Sciences). Following washing, the membranes were incubated for 1 h with the secondary HRP–linked horse-anti-mouse antibody (Cell Signaling Technology). Immunoblotted proteins were visualised by SuperSignal West Dura Substrate (PIERCE ThermoScientific) using LI-COR Odyssey Fc Imager (LI-COR Biosciences) and Image studio Vr 2.0 software.

### ATP and IL-33 release

ATP and IL-33 were measured in cell supernatants using an ATP assay from BioThema (Sweden) or a DuoSet ELISA from R&D Systems (Abingdon, UK) according to the manufacturer’s descriptions, respectively.

### Statistical analysis

Data were analysed using the software GraphPad Prism version 6.0 (San Diego, CA, USA) and expressed as mean value with SEM. Significant variations between paired groups were determined by the non-parametric tests Friedman´s one-way ANOVA and the Wilcoxon matched-pairs signed ranks test, while the Mann–Whitney test was used to analyse differences between unpaired groups. P values of less than 0.05 were considered statistically significant.

## Results

### Conditioned media from bronchial epithelial cells trigger expression of IL-33 in BSMCs and concurrent stimulation with dsRNA enhances the response

**Fig. 1:**
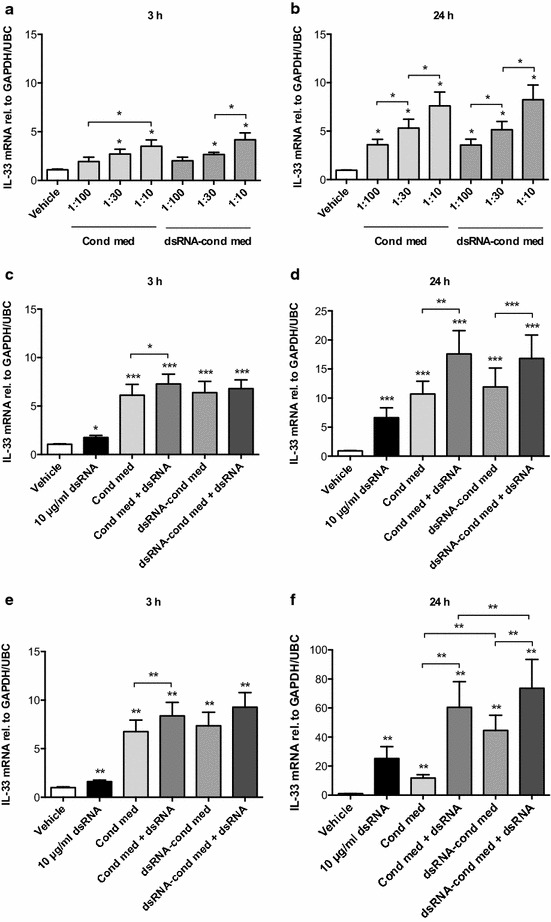
Bronchial smooth muscle cell (BSMC) expression of IL-33 is induced by epithelial conditioned media and further enhanced by dsRNA. To establish an optimal concentration of epithelial conditioned media, BSMCs from healthy subjects were stimulated with different dilutions of conditioned media from either non-treated (cond med) or dsRNA-treated (dsRNA-cond med) bronchial epithelial cells and mRNA expression of IL-33 was analysed by RT-qPCR after 3 and 24 h (**a**, **b**). IL-33 expression after stimulation with 10 μg/ml dsRNA alone or in combination with epithelial conditioned media was analysed in both healthy (**c**, **d**) and asthmatic (**e**, **f**) BSMCs. Epithelial basal medium diluted 1:10 in BSMC medium was used as vehicle control. Data are presented as mean with SEM and n = 3–6 independent experiments from three BSMC donors. *p ≤ 0.05, **p ≤ 0.01 and ***p ≤ 0.001 compared to vehicle if not otherwise indicated

To address our hypothesis that epithelial mediators may induce expression of IL-33 in BSMCs, we stimulated BSMCs with conditioned media obtained from non-treated or dsRNA-treated bronchial epithelial cells. Concentration-dependent effects on IL-33 mRNA expression were induced in healthy BSMCs at 3 h (Fig. 1a) and further augmented at 24 h (Fig. 1b). Healthy BSMCs were also stimulated directly with dsRNA, either alone or in combination with epithelial conditioned media in an optimal concentration of 1:10. dsRNA alone induced modest expression of IL-33 mRNA at 3 h (Fig. 1c), which was more pronounced at 24 h (Fig. 1d). At 24 h, direct stimulation of BSMCs with dsRNA in combination with conditioned media from either non-treated or dsRNA-treated epithelial cells caused an additive induction of IL-33 expression (Fig. 1d). Since BSMCs are known to be in juxtaposition with the bronchial epithelium in asthma, we investigated the effect of epithelial conditioned media also on BSMCs from asthmatic subjects. Similar to in healthy BSMCs, IL-33 expression was induced by dsRNA and/or conditioned media at 3 h (Fig. 1e) and further increased at 24 h (Fig. 1f). Although no statistical significance could be achieved, the observed IL-33 responses at 24 h tended to be more pronounced in BSMCs from asthmatic subjects (Fig. 1f) compared with healthy BSMCs (Fig. 1d). Interestingly however, in contrast to the observed effect in healthy BSMCs (Fig. 1a–d), IL-33 expression in asthmatic BSMCs at 24 h was more increased after stimulation with conditioned medium from dsRNA-treated epithelial cells compared to conditioned medium from non-treated epithelial cells (Fig. 1f).

### IL-33 is produced by BSMCs in response to specific ligands for TLR3 and RIG-I-like receptors

**Fig. 2:**
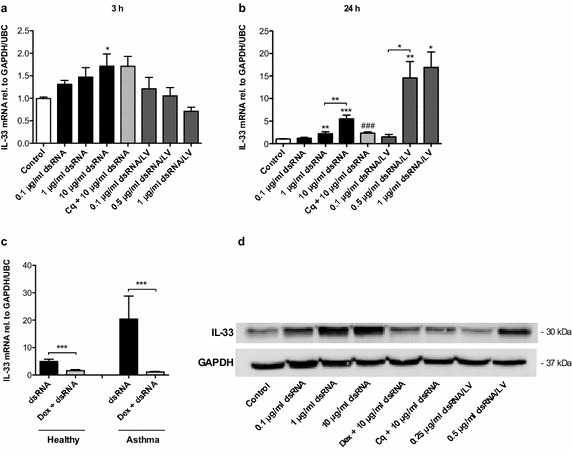
Specific ligands for TLR3 and RIG-I-like receptors trigger production of IL-33 in BSMCs. BSMCs from healthy subjects were stimulated with different concentrations of dsRNA and dsRNA/LyoVec (dsRNA/LV). IL-33 mRNA expression (**a**, **b**) was analysed after 3 and 24 h by RT-qPCR and IL-33 protein was determined by western blot after 24 h (**d**). Cells were also pretreated with 10 μg/ml of the TLR3 inhibitor chloroquine (Cq) (**a**, **b** and **d**). The effect of 1 μg/ml dexamethasone (Dex) on 10 μg/ml dsRNA-induced IL-33 expression was evaluated in healthy (**c**, **d**) and asthmatic BSMCs (**c**) after 24 h stimulation. Data are presented as mean with SEM and n = 6 independent experiments from three BSMC donors. *p ≤ 0.05, **p ≤ 0.01 and ***p ≤ 0.001 compared to control if not otherwise indicated and ^###^ *p* compared to 10 μg/ml dsRNA. A representative western blot image of IL-33 protein is shown

We have previously shown that BSMCs similar to bronchial epithelial cells express TLR3 and RIG-I-like receptors and that stimulation of these PRRs leads to significant BSMC production of antiviral interferons [28]. As demonstrated above (Fig. 1b–d), dsRNA also induced IL-33 expression in both healthy and asthmatic BSMCs. To further investigate a potential role of TLR3 and RIG-I-like receptors in BSMC production of IL-33, healthy BSMCs were stimulated with different doses of dsRNA and dsRNA/LyoVec, primarily activating TLR3 and RIG-I-like receptors respectively. At 3 h a modest IL-33 expression was only induced by 10 μg/ml dsRNA (Fig. 2a), but at 24 h both dsRNA and dsRNA/LyoVec concentration-dependently increased expression of IL-33 mRNA as well as IL-33 protein (Fig. 2b, d). The TLR3 inhibitor chloroquine reduced dsRNA-induced IL-33 expression (Fig. 2b, d). Further, we found that dexamethasone inhibited dsRNA-induced IL-33 expression in BSMCs from both healthy (Fig. 2c, d) and asthmatic subjects (Fig. 2c).

### RV infection triggers production of IL-33 by BSMCs

**Fig. 3:**
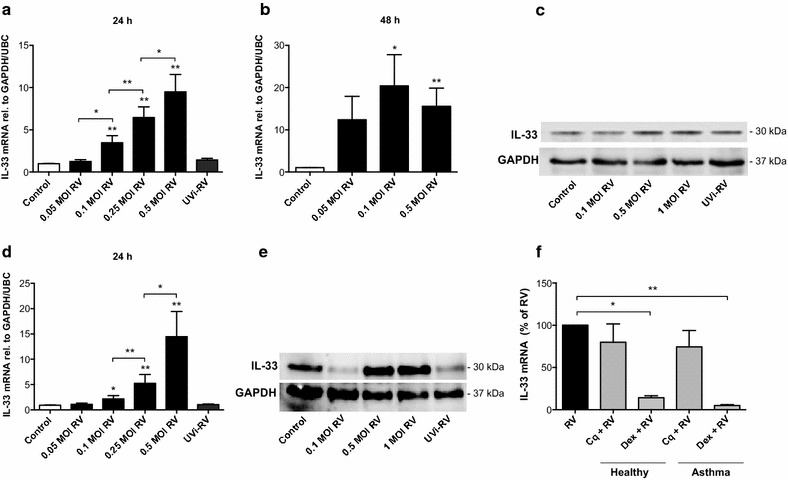
IL-33 is produced by BSMCs upon infection with RV. BSMCs from healthy (**a**–**c**) and asthmatic subjects (**d**, **e**) were infected with RV at different doses (*MOI* multiplicity of infection). Expression of IL-33 mRNA and protein were analysed by RT-qPCR 24 and 48 h post infection and by western blot 24 h post infection. UVi-RV = UV inactivated RV corresponding to the highest dose of RV used. The effects of chloroquine (10 μg/ml) and dexamethasone (1 μg/ml) on RV-induced (0.5 MOI) IL-33 expression were evaluated in healthy and asthmatic BSMCs 24 h post infection (**f**). Data are presented as mean with SEM and n = 3–6 independent experiments from three BSMC donors. *p ≤ 0.05 and **p ≤ 0.01 compared to control if not otherwise indicated. Representative western blot images of IL-33 protein are shown

Next, we infected BSMCs with RV. RV infection induced a dose-dependent increase of IL-33 mRNA in BSMCs from healthy individuals at 24 h (Fig. 3a) being further enhanced 48 h post infection (Fig. 3b). Increased IL-33 protein also occurred (Fig. 3c). Further, IL-33 was also significantly induced in RV-infected BSMCs from asthmatic donors at 24 h (Fig. 3d, e). Release of low levels of IL-33 protein was detected from RV-infected BSMCs only and occurred in parallel with RV-induced cytopathic effects (unpublished observations). Infection with UV-irradiated RV did not elicit an IL-33 response (Fig. 3a, c–e), suggesting that RV-induced IL-33 is replication dependent. Chloroquine tended to reduce RV-induced IL-33 expression proposing involvement of TLR3 signalling (Fig. 3f). Similar to dsRNA-induced IL-33, dexamethasone inhibited RV-induced IL-33 expression in BSMCs from both healthy and asthmatic individuals (Fig. 3f).

### IL-33 induced by viral stimulation and epithelial condition medium in BSMCs is blocked by suramin, a broad inhibitor of purinergic signalling

**Fig. 4:**
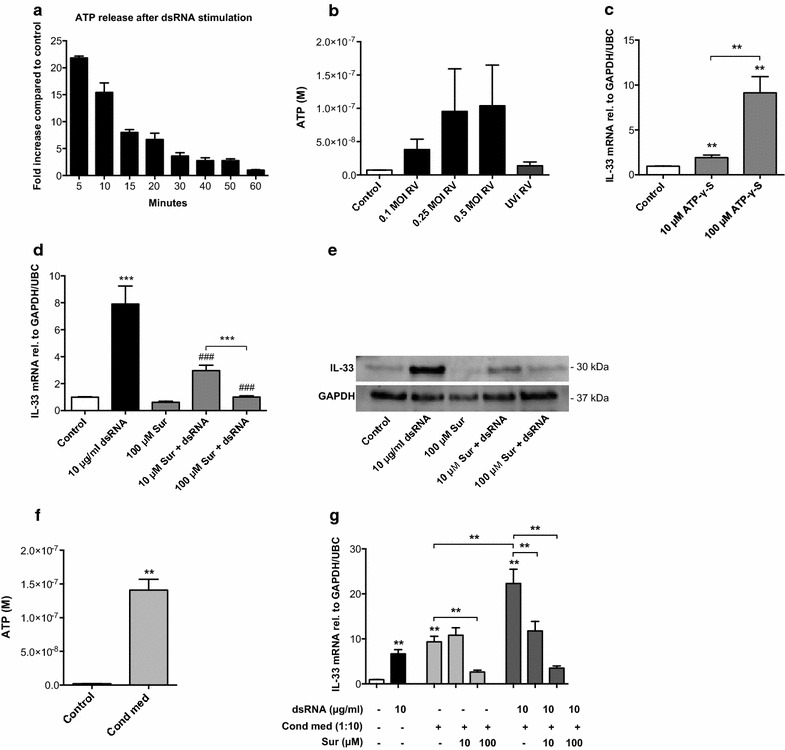
Blocking purinergic signalling inhibits BSMC expression of IL-33 induced by dsRNA and epithelial condition medium. BSMCs were stimulated with 10 μg/ml dsRNA for 5–60 min and ATP was analysed in cell supernatants and data related to that of control samples (**a**). ATP release was also measured in BSMC supernatants 24 h after RV-infection (**b**). BSMC expression of IL-33 mRNA was analysed by RT-qPCR 24 h after stimulation with non-hydrolysable ATP (ATP-γ-S) (**c**). The effect of the purinergic inhibitor suramin (Sur) on dsRNA-induced IL-33 expression was evaluated at both mRNA (**d**) and protein (**e**) levels with RT-qPCR and western blot respectively. ATP was measured in bronchial epithelial conditioned medium (n = 4 donors) and compared to non-conditioned medium (control) (**f**) and the effect of suramin on conditioned medium-induced IL-33 mRNA (**g**) was analysed 24 h after stimulation. Data are presented as mean with SEM and n = 2–6 independent experiments from two or three BSMC donors. *p ≤ 0.05, **p ≤ 0.01, and ***p ≤ 0.001 compared to control if not otherwise indicated and ^###^ *p* ≤ 0.001 compared to 10 μg/ml dsRNA. A representative western blot image of IL-33 is shown

When analysing BSMC supernatants we found that dsRNA induces immediate release of ATP above baseline levels, peaking 5 min or earlier after stimulation (Fig. 4a). We also found increased levels of ATP in supernatants from RV-infected BSMCs compared to control cells (Fig. 4b). To test if ATP could induce IL-33 expression in BSMCs, we stimulated the cells with non-hydrolysable ATP (ATP-γ-S) and found increased IL-33 mRNA after 24 h (Fig. 4c). To further show that ATP is involved in dsRNA-induced IL-33 production we treated BSMCs with suramin, a broad inhibitor of purinergic signalling. We found concentration-dependent inhibition by suramin of the dsRNA-induced IL-33 expression at both mRNA (Fig. 4d) and protein levels (Fig. 4e). We then analysed the ATP content in bronchial epithelial conditioned medium and found a significantly higher concentration of ATP compared to non-conditioned medium (Fig. 4f). Further, when suramin was added to BSMCs prior to bronchial epithelial conditioned medium, IL-33 expression in BSMCs was significantly reduced (Fig. 4g).

## Discussion

Recent evidence highlights that BSMCs are not only responsible for regulating bronchomotor tone, but have many regulatory functions related to inflammation and innate immunity in the airways [26, 27]. In the present study we report for the first time that IL-33 production is increased in healthy and asthmatic BSMCs upon activation of TLR3 and RIG-I-like receptors as well as after RV infection and that this response is sensitive to corticosteroid treatment. We also show that BSMC expression of IL-33 is induced by factor(s) released by bronchial epithelial cells and further augmented by rhinoviral stimuli. Moreover, our results suggest that the cellular metabolite ATP is involved in mediating IL-33 expression since this mediator is produced upon RV infection and TLR3 activation, elevated in epithelial conditioned medium and pharmacological modulation involving blocking of purinergic receptors reduces the present IL-33 induction in BSMCs.

To our knowledge only one study, recently published by Jackson and colleagues, has reported RV infection-induced IL-33 production by human airway cells, and that study involved primary bronchial epithelial cells [25]. Although the bronchial epithelium is the main target for RV, infection also of submucosal cells has been reported in situ in diseased lower airways possibly reflecting a compromised epithelial barrier occurring in asthma and COPD [30, 31]. Here we demonstrate that IL-33 is produced upon RV infection of primary human BSMCs from healthy and asthmatic subjects. Since UV-irradiated RV did not induce IL-33 this effect is likely to be replication dependent. In agreement with involvement of the biologically active intermediate dsRNA, chloroquine, an agent that inhibits endosomal TLR3 activation [32], partly reduced the present IL-33 response to RV. Further, by employment of agonists primarily activating either TLR3 (dsRNA) or RIG-I-like receptors (dsRNA/LyoVec), we demonstrated that both these RV infection-mimicking PRR ligands induced IL-33 expression in BSMCs. The discrepancies between the mRNA and protein data comparing the two ligands at 24 h may be explained by different receptor-ligand kinetics as observed in our previous study where stronger responses with dsRNA/LyoVec occurred later than with dsRNA and corresponded with upregulated expression of RIG-I and MDA5 [28]. Our data on RIG-I-like receptor-induced IL-33 is in accord with a previous study by Polumuri and colleagues who observed particularly robust transcription of IL-33 in murine macrophages upon stimulation with RIG-I-like receptor agonists [22]. Since the effect of the TLR3 inhibitor chloroquine on RV-induced IL-33 was only partial, our data further implies a role also for cytosolic RIG-I-like receptors in RV-induced IL-33 production in BSMCs. However, future studies are needed to address this question more in detail.

Préfontaine and colleagues have previously described airway smooth muscle cell overexpression of IL-33 in endobronchial biopsy specimens from severe asthmatics [7]. In asthma, especially in the severe state, the airway smooth muscle also exhibits extensive remodelling including proliferation and migration leading to increased smooth muscle mass [33, 34]. Such alterations may position BSMCs in closer proximity to the airway epithelial lining potentially enhancing epithelial-smooth muscle crosstalk. This possibility supports a potential role of the present finding that factors spontaneously released by bronchial epithelial cells increased IL-33 expression in BSMCs. Hence, epithelium-derived factors may in part be responsible for baseline expression of IL-33 in juxtapositioned bronchial smooth muscle. Interestingly, asthmatic BSMCs exhibited even more pronounced IL-33 expression after stimulation with conditioned medium derived from epithelial cells treated with dsRNA. Although the molecular mechanism underlying this finding is currently unclear, the observation that activation of PRRs in BECs enhance IL-33 production in BSMCs from asthmatic but not healthy subjects is intriguing and merits further investigation in future studies. Moreover, concurrent additional stimulation of BSMCs with dsRNA further increased all responses to epithelial conditioned media, proposing that epithelial-induced IL-33 expression in BSMCs may be potentiated by exogenous microbial or endogenous injury-associated TLR3 ligands, such as dsRNA. The somewhat surprising discovery that epithelial cells already at baseline produce factors that increase BSMC IL-33 expression needs follow-up by future studies. A range of epithelial challenges can be listed, including the present viral stimuli, as candidates that potentially could increase the influence of epithelial cells on BSMC expression and production of IL-33.

In the present study we observed that asthmatic BSMCs in comparison to healthy cells tended to have an elevated IL-33 response after stimulation with epithelial conditioned media and dsRNA, and possibly after RV infection too. However, a larger number of study subjects would be required to investigate if statistically significant disease-related differences exist. Further, our observation that viral-induced IL-33 expression in BSMCs from healthy as well as asthmatic subjects was sensitive to dexamethasone treatment contrasts to the study by Préfontaine and colleagues where the same corticosteroid failed to reduce the transcription of IL-33 in TNF-α-stimulated airway smooth muscle cells [7]. These discrepancies may in part relate to the nature of the stimuli or the smooth muscle cell population studied. Chang and colleagues recently reported that airway smooth muscle cells from severe asthmatics have impaired nuclear translocation of the glucocorticoid receptor, which may provide one explanation to the corticosteroid insensitivity found in these patients [35]. Whether or not the effect of corticosteroids on viral-induced IL-33 is compromised in BSMCs from severe asthmatics is something to consider for future studies.

Extracellular ATP in the airways has been implicated in both asthma and COPD pathogenesis [14–17]. Studies also show that release of ATP into the extracellular space upon exposure to allergen, smoke or viral infections may promote an inflammatory microenvironment [14, 15, 17, 18, 36]. In the present study extracellular ATP levels were increased rapidly after stimulation of BSMCs with dsRNA and elevated in supernatants from RV-infected BSMCs. By pretreating BSMCs with suramin, a broad-spectrum purinergic receptor antagonist, we could further demonstrate that IL-33 expression in response to dsRNA was abrogated in a concentration-dependent manner, implying a role for ATP signalling. Recently, Kouzaki and colleagues reported that exposure of primary human bronchial epithelial cells to the fungal allergen *AlternariaAlternata* leads to noncytotoxic release of pre-formed IL-33 and proposed that this response involved ATP release and subsequent stimulation of purinergic P2 receptors [13]. However, to our knowledge, our observation on involvement of ATP in TLR3-mediated enhanced IL-33 production is novel.

Based on our observations including the finding that exogenous ATP could trigger BSMC expression of IL-33, we speculated that ATP released from bronchial epithelial cells also could be involved in the enhanced IL-33 expression found in BSMCs. In support of this hypothesis, we could detect significantly higher levels of ATP in conditioned medium from bronchial epithelial cells compared to non-conditioned epithelial medium. Further, pretreatment of BSMCs with the broad P2R-antagonist suramin reduced IL-33 expression in response to both epithelial conditioned medium alone and to the combination of conditioned medium and dsRNA. Thus, our findings suggest the involvement of ATP, likely in concert with other epithelial-derived mediators, in the enhanced IL-33 expression observed in BSMCs challenged with bronchial epithelial conditioned media.

## Conclusions

In summary, in this study we demonstrate BSMC production of the immunoregulatory cytokine IL-33, which has been forwarded as a potential disease factor in asthma. Further, we show that conditioned media from bronchial epithelial cells as well as RV infection and stimulation with viral infection mimics acting on TLR3 and RIG-I-like receptors increase IL-33 in BSMCs from both healthy and asthmatic individuals. The present data suggest that epithelial mediators may induce baseline BSMC IL-33 gene expression and that augmented expression and production of IL-33 are caused by RV infection of BSMCs and stimulation of TLR3 and RIG-I-like receptors. We further suggest that ATP is involved as an epithelial-derived mediator of BSMC IL-33 expression and, similarly, the ATP/P2R-axis may be involved in the present viral stimuli-induced effects directly on BSMCs.

## References

[1] Schmitz J, Owyang A, Oldham E, Song Y, Murphy E, McClanahan TK (2005). IL-33, an interleukin-1-like cytokine that signals via the IL-1 receptor-related protein ST2 and induces T helper type 2-associated cytokines. Immunity.

[2] Hayakawa H, Hayakawa M, Kume A, Tominaga S (2007). Soluble ST2 blocks interleukin-33 signaling in allergic airway inflammation. J Biol Chem.

[3] Kearley J, Buckland KF, Mathie SA, Lloyd CM (2009). Resolution of allergic inflammation and airway hyperreactivity is dependent upon disruption of the T1/ST2-IL-33 pathway. Am J Respir Crit Care Med.

[4] Carriere V, Roussel L, Ortega N, Lacorre DA, Americh L, Aguilar L (2007). IL-33, the IL-1-like cytokine ligand for ST2 receptor, is a chromatin-associated nuclear factor in vivo. Proc Natl Acad Sci USA..

[5] Moussion C, Ortega N, Girard JP (2008). The IL-1-like cytokine IL-33 is constitutively expressed in the nucleus of endothelial cells and epithelial cells in vivo: a novel ‘alarmin’?. PLoS One.

[6] Prefontaine D, Nadigel J, Chouiali F, Audusseau S, Semlali A, Chakir J (2010). Increased IL-33 expression by epithelial cells in bronchial asthma. J Allergy Clin Immunol..

[7] Prefontaine D, Lajoie-Kadoch S, Foley S, Audusseau S, Olivenstein R, Halayko AJ (2009). Increased expression of IL-33 in severe asthma: evidence of expression by airway smooth muscle cells. J Immunol..

[8] Liu X, Li M, Wu Y, Zhou Y, Zeng L, Huang T (2009). Anti-IL-33 antibody treatment inhibits airway inflammation in a murine model of allergic asthma. Biochem Biophys Res Commun..

[9] Bunting MM, Shadie AM, Flesher RP, Nikiforova V, Garthwaite L, Tedla N (2013). Interleukin-33 drives activation of alveolar macrophages and airway inflammation in a mouse model of acute exacerbation of chronic asthma. Biomed Res Int..

[10] Moffatt MF, Gut IG, Demenais F, Strachan DP, Bouzigon E, Heath S (2010). A large-scale, consortium-based genomewide association study of asthma. N Engl J Med.

[11] Torgerson DG, Ampleford EJ, Chiu GY, Gauderman WJ, Gignoux CR, Graves PE (2011). Meta-analysis of genome-wide association studies of asthma in ethnically diverse North American populations. Nat Genet.

[12] Haraldsen G, Balogh J, Pollheimer J, Sponheim J, Kuchler AM (2009). Interleukin-33—cytokine of dual function or novel alarmin?. Trends Immunol.

[13] Kouzaki H, Iijima K, Kobayashi T, O’Grady SM, Kita H (2011). The danger signal, extracellular ATP, is a sensor for an airborne allergen and triggers IL-33 release and innate Th2-type responses. J Immunol..

[14] Idzko M, Hammad H, van Nimwegen M, Kool M, Willart MA, Muskens F (2007). Extracellular ATP triggers and maintains asthmatic airway inflammation by activating dendritic cells. Nat Med.

[15] Muller T, Vieira RP, Grimm M, Durk T, Cicko S, Zeiser R (2011). A potential role for P2X7R in allergic airway inflammation in mice and humans. Am J Respir Cell Mol Biol.

[16] Esther CR, Lazaar AL, Bordonali E, Qaqish B, Boucher RC (2011). Elevated airway purines in COPD. Chest.

[17] Lommatzsch M, Cicko S, Muller T, Lucattelli M, Bratke K, Stoll P (2010). Extracellular adenosine triphosphate and chronic obstructive pulmonary disease. Am J Respir Crit Care Med.

[18] Idzko M, Ferrari D, Eltzschig HK (2014). Nucleotide signalling during inflammation. Nature.

[19] Johnston SL, Sanderson G, Pattemore PK, Smith S, Bardin PG, Bruce CB (1993). Use of polymerase chain reaction for diagnosis of picornavirus infection in subjects with and without respiratory symptoms. J Clin Microbiol.

[20] Leung TF, To MY, Yeung AC, Wong YS, Wong GW, Chan PK (2010). Multiplex molecular detection of respiratory pathogens in children with asthma exacerbation. Chest.

[21] Kawai T, Akira S (2006). Innate immune recognition of viral infection. Nat Immunol.

[22] Polumuri SK, Jayakar GG, Shirey KA, Roberts ZJ, Perkins DJ, Pitha PM (2012). Transcriptional regulation of murine IL-33 by TLR and non-TLR agonists. J Immunol..

[23] Zhang L, Lu R, Zhao G, Pflugfelder SC, Li DQ (2011). TLR-mediated induction of pro-allergic cytokine IL-33 in ocular mucosal epithelium. Int J Biochem Cell Biol.

[24] Sponheim J, Pollheimer J, Olsen T, Balogh J, Hammarstrom C, Loos T (2010). Inflammatory bowel disease-associated interleukin-33 is preferentially expressed in ulceration-associated myofibroblasts. Am J Pathol.

[25] Jackson DJ, Makrinioti H, Rana BM, Shamji BW, Trujillo-Torralbo MB, Footitt J (2014). IL-33-dependent Type 2 inflammation during rhinovirus-induced asthma exacerbations in vivo. Am J Respir Crit Care Med.

[26] Damera G, Tliba O, Panettieri RA (2009). Airway smooth muscle as an immunomodulatory cell. Pulm Pharmacol Ther.

[27] Koziol-White CJ, Panettieri RA (2011). Airway smooth muscle and immunomodulation in acute exacerbations of airway disease. Immunol Rev.

[28] Calven J, Yudina Y, Uller L (2013). Rhinovirus and dsRNA induce RIG-I-like receptors and expression of interferon β and λ1 in human bronchial smooth muscle cells. PLoS One.

[29] Brandelius A, Yudina Y, Calven J, Bjermer L, Andersson M, Persson C (2011). dsRNA-induced expression of thymic stromal lymphopoietin (TSLP) in asthmatic epithelial cells is inhibited by a small airway relaxant. Pulm Pharmacol Ther.

[30] Papadopoulos NG, Bates PJ, Bardin PG, Papi A, Leir SH, Fraenkel DJ (2000). Rhinoviruses infect the lower airways. J Infect Dis.

[31] Wos M, Sanak M, Soja J, Olechnowicz H, Busse WW, Szczeklik A (2008). The presence of rhinovirus in lower airways of patients with bronchial asthma. Am J Respir Crit Care Med.

[32] de Bouteiller O, Merck E, Hasan UA, Hubac S, Benguigui B, Trinchieri G (2005). Recognition of double-stranded RNA by human toll-like receptor 3 and downstream receptor signaling requires multimerization and an acidic pH. J Biol Chem.

[33] Joubert P, Hamid Q (2005). Role of airway smooth muscle in airway remodeling. J Allergy Clin Immunol..

[34] Takeda N, Sumi Y, Prefontaine D, Al Abri J, Al Heialy N, Al-Ramli W (2009). Epithelium-derived chemokines induce airway smooth muscle cell migration. Clin Exp Allergy.

[35] Chang PJ, Michaeloudes C, Zhu J, Shaikh N, Baker J, Chung KF (2015). Impaired nuclear translocation of the glucocorticoid receptor in corticosteroid-insensitive airway smooth muscle in severe asthma. Am J Respir Crit Care Med.

[36] Esther CR, Alexis NE, Clas ML, Lazarowski ER, Donaldson SH, Ribeiro CM (2008). Extracellular purines are biomarkers of neutrophilic airway inflammation. Eur Respir J.

